# Impact of Raltegravir on immune reconstitution and thymopoiesis in HIV-1-infected patients with undetectable viremia

**DOI:** 10.1186/1758-2652-13-S3-O12

**Published:** 2010-11-04

**Authors:** C Garrido, N Rallón, N Zahonero, M de la O López, V Soriano, C de Mendoza, JM Benito

**Affiliations:** 1 Infectious Diseases, Hospital Carlos III, Madrid, Spain

## Background

CD4 gains under antiretroviral treatment might come from cells recently migrated from the thymus or from cells proliferating in the periphery. CD31 on the surface of CD4+ T cells has been shown to identify recent thymic emigrants. We evaluated the characteristics of the CD4 gain observed in patients switching to a Raltegravir (RAL) containing regimen.

## Methods

Patients on highly active antiretroviral therapy (HAART) with long-term suppressed viremia were selected and divided according to treatment: switched to RAL vs. maintained the same regimen (control group). Samples were collected at the time of RAL switch (0) and six months after (+6). Immune parameters were assessed in peripheral blood mononuclear cells (PBMCs) using five-colour flow cytometry. Analysis of naïve and memory CD4+ Tcells (CD45RA and CD27) was combined with measurements of activation (CD38) and with CD31 expression. Results were reported as median [IQR] and comparisons were performed with Mann-Whitney tests.

## Results

Thirty-seven patients (19 RAL and 18 control) were selected. Baseline characteristics did not differ among groups for CD4+ T cell count (322 cells/mm^3^ [204-499]) and percentage (18% [12.5-26.5]), age (44 years old [41.5-48]) and time with suppressed viremia (2.12 years [0.84-4.80]).

Six months after switching to RAL, CD4+ T cell count was significantly higher than baseline (**448** [288-575] vs. **322** [242-594] cells/mm^3^, p=0.026), while patients who did not switch to RAL maintained stable CD4 values (**330** [176-425] vs. **312** [141-478], p=0.813).

No differences were observed from 0 to +6 in any group for immune activation or CD31 expression. However, in the RAL group, the proportion of naïve CD4+ T cells increased six months after the switch (from **18.2%** [9.3-29.3] to **23.7%** [12.7-31.4], p=0.014), while the level of effector memory CD4+ T cells experienced a significant decrease (**13.3%** [9-21.3] vs. **10.2%** [5.5-16.8], p=0.005). All these subsets remained stable in the control group during the follow up.

## Conclusions

Switching to a RAL-containing regimen induced a significant gain in CD4+ T cell count compared with patients who maintained the same treatment. This improvement was mainly due to an increase in CD4 naïve cells. However, it could not be explained by an increase of the thymic function, suggesting that CD4 gains under RAL treatment may come from peripheral expansion.

